# A new mathematical model and experimental validation on foamy-oil flow in developing heavy oil reservoirs

**DOI:** 10.1038/s41598-017-08882-2

**Published:** 2017-08-17

**Authors:** Pengcheng Liu, Zhenbao Mu, Wenhui Li, Yongbin Wu, Xiuluan Li

**Affiliations:** 1 School of Energy Resources, China University of Geosciences, 29 Xueyuan Road, Beijing, 100083 China; 2 Research Institute of Petroleum Exploration and Development, SINOPEC, 29 Xueyuan Road, Beijing, 100083 China; 3 Research Institute of Petroleum Exploration and Development, PetroChina, 31 Xueyuan Road, Beijing, 100083 China

## Abstract

To model foamy-oil flow in the development of heavy oil reservoirs, three depletion experiments were conducted with foamy oil treated as a pseudo-single-phase flow. In this pseudo single phase, dispersed bubbles are viewed as a part of the oil, and the redefined effective permeability varies with the changes of pressure depletion rate, oil viscosity, and gas saturation. A mathematical expression for the effective permeability was developed based on experiments, where the viscosity of foamy oil is assumed to be approximately equal to the saturated oil under equivalent conditions. The compressibility coefficient of foamy oil is treated as a volume-weighted compressibility coefficient of that of oil and gas phases. A new mathematical model for foamy-oil flow was proposed with consideration of foamy-oil supersaturation. To validate the mathematical model, the oil recovery and the production gas-oil ratio (GOR) calculated by the new model, conventional black oil model, supersaturation model and pseudo-bubble-point (PBP) model were all compared with those of the experimental data. The new model provided a substantially improved fit to the experimental data compared with the rest three models, which verifies the suitability of the mathematical model presented for simulating foamy-oil flow in the development of heavy oil reservoirs.

## Introduction

Foamy oil is a term used to describe a type of unconventional heavy oil where gas bubbles are dispersed in a continuous oil phase^1–3^. This type of oil is often found in the primary depletion of some heavy oil reservoirs in Canada, Venezuela, China and Oman, which have shown anomalously high production performances such as much higher production oil rate and primary recovery, and a lower production gas-oil ratio (GOR), compared with conventional heavy oil reservoirs^4–7^.

Taking advantage of foamy oil’s properties is a much more cost-effective approach compared with other methods to develop heavy oil reservoirs, like steam flooding. Therefore, making it clear that the mechanisms of foamy oil flowing in porous media has great economic benefits. Establishment of a reasonable mathematical model for foamy-oil flow is the basis and premise of numerical simulation to develop heavy oil reservoirs. Initial numerical simulations of foamy-oil flow in porous media often employed the conventional black oil model by adjusting various reservoir and fluid parameters such as the reservoir permeability, the fluid viscosity, and the relative permeability curves of the oil and gas phases^8–11^. Although simulations based on the conventional black oil model probably have agreed well with the well production history, the characteristics of foamy-oil flow were not captured, and the predictions of future scenarios by the simulations were obscure.

Due to the unique properties of foamy oil in porous media, the conventional black oil model is not suitable^12–16^. Several researchers have therefore studied the specific mechanisms of foamy-oil flow, and proposed several mathematical models. It is believed that one of the reasons for the anomalously high production performance is owing to the displacement pressure provided by the strong compressibility of foamy oil in addition to a delay in the formation of a continuous gas phase during oil production^5^. The majority of models applicable to foamy oil are modified conventional solution gas drive models based on foamy-oil characteristics and experimental data. Smith (1988) appears to be the first to consider foamy oil, where a pseudo-pressure form derived from Darcy’s law and a radial diffusivity equation were used to simulate the pressure behavior of foamy-oil wells. Nevertheless, the model addressed the characteristics of foamy oil too simply^17^. Fractional-flow models were applied to match the production performance of foamy-oil wells through modifying a suitable fractional-flow curve from the relative permeabilities of gas and oil^18–20^. However, obtaining a realistic fractional-flow curve has certain difficulties and requires constant trial and error. Non-equilibrium models assume that all the gas evolved from the solution is initially dispersed in the oil^21–25^. The transformation from gases in solution to dispersed bubbles, and from dispersed bubbles to a continuous gas phase were both described as sequential-rate processes. These models reflect an important feature of foamy oil, in which its characteristics undergo time-dependent changes, but the models are not valid for predicting the outcome of an evolving scenario under varying flow conditions. Pseudo-bubble-point (PBP) models assume that the continuous gas phase begins to form until the formation pressure is below the pseudo-bubble-point pressure, which is defined as an adjustable parameter in the description of this property of foamy oil^26, 27^. Though this type of model can capture some important characteristics of foamy-oil flow in porous media, it cannot reflect its time-dependent changes. Supersaturation model assumes that the flow rate of foamy oil follows Darcy’s law, and established a relaxation time for the system returning to an equilibrium state^24, 28^. However, this model incorporates numerous empirically determined parameters and brings a lot of uncertainties of simulation results. Hypothetical models of foamy-oil flow in porous media have been developed as well, where micro-bubbles are assumed to be coated with asphaltenes, preventing the growth and coalescence of bubbles, and resulting in a dramatically reduced oil viscosity^29–31^. Similarly, it has been suggested that oil mobility is improved by bubble nucleation at the grain surface owing to lubrication effects^32–35^. However, neither of these assumptions have yet been supported by direct experimental evidence.

In brief, previous mathematical models can offer some important information of foamy-oil reservoirs, but they also have their own drawbacks more or less. Therefore, the objective of this paper is to develop a more suitable mathematical model for foamy-oil flow. We have adopted the concept of the pseudo-bubble-point pressure^26, 27^, the relaxation time parameter, which is a description of the supersaturation phenomenon^24, 28, 36^. This model combines the advantages of both the pseudo-bubble-point (PBP) models and supersaturation models, and it overcomes their disadvantages to some extents. As a consequence, the model proposed in this paper can describe the properties more accurate, and the calculation of some crucial parameters is more closed to the experimental data than the previous models that we mentioned above. It indicated that this model is an advanced one and it is more suitable than other models to simulate the development of foamy oils.

It must be pointed that we only confine our discussion about the primary depletion process on foamy-oil flow for heavy oil reservoirs. For secondary or/and tertiary flooding by injected surfactants or/and other agents, as well as thermal energy, a compositional model is required and they are not the focus of this paper.

## Experiments

This experimental part has two functions, the first one is to help establish a mathematical expression of foamy-oil relative permeability, in which the effective permeability was measured through experiments, and the second function is to help validate the accuracy of the model we proposed in this paper by comparing the recovery and production gas-oil ratio (GOR) of experiments with the new model and the other three models which have been proposed by other researchers.

### Experimental apparatus

**Figure 1:**
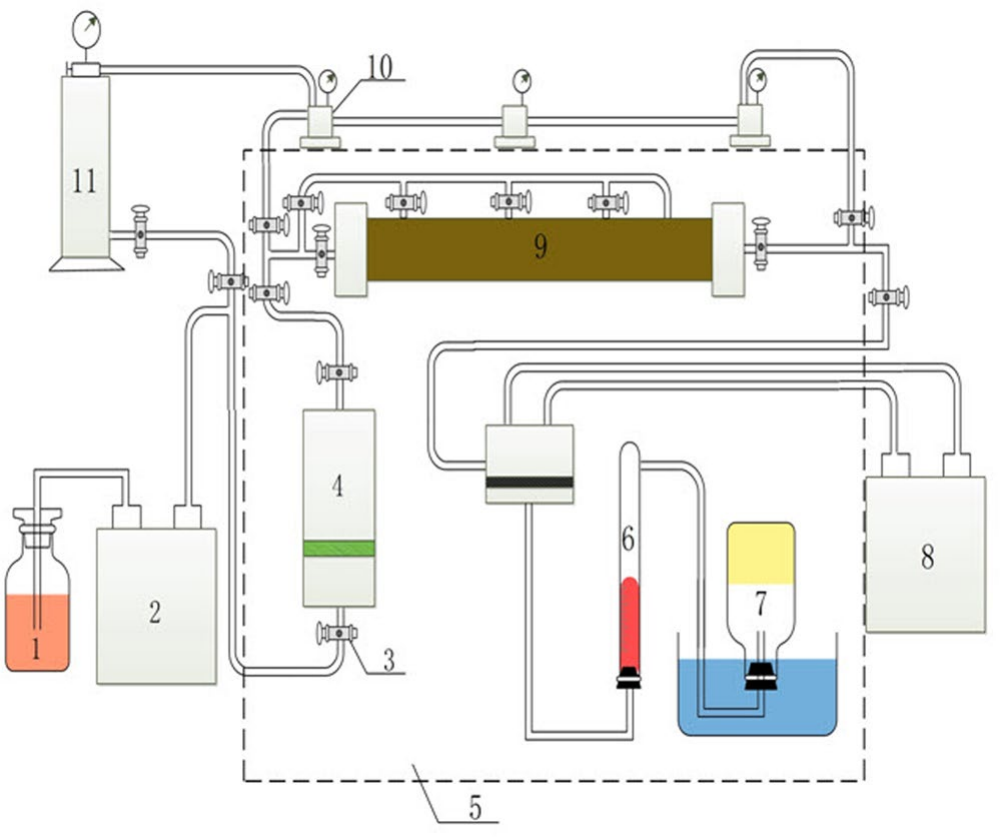
Schematic diagram of the apparatus employed for experimental evaluation of foamy-oil flow. Note: 1-pump working fluid; 2-ISCO pump; 3-valve; 4-sample container; 5-thermostatic system; 6-oil volume gauge; 7-gas volume gauge; 8-back pressure pump; 9-long sand pack; 10-six-port valve; 11-intermediate container.

Figure 1 illustrated the equipment used in the laboratory depletion experiments. An intermediate container is used to transfer the saturated oil sample from the sample container to the long sand pack. The long sand pack is constructed from a special rough-walled steel tube of 5.0 cm diameter and 80.0 cm length with a maximum burden pressure of 32.0 MPa. A back pressure pump with a maximum operating pressure of 70.0 MPa is used to control the pressure of the outlet of the sand pack. An International Standard Classification of Occupations (ISCO) pump is used for driving the fluid into the sand pack and for pressurizing the system at a constant pressure or constant flow rate. The maximum flow rate is 25.0 ml/min and the maximum pressure is 70.0 MPa. An electronically controlled thermostat is used to ensure a constant temperature in the range of room temperature to 200.0 °C. A metering system including gas and oil volume gauges is used to record the amount of gas and oil production. A vacuum pump is used to initialize the sand pack to the designated pressure at the beginning of the experiments.

### Material

The design of sand packs used in the experiments were according to the properties of layers in MPE-3 Block in Venezuela, the sand packs were comprised of clean quartz sand, and the compressibility coefficient was 6.5 × 10^−4^ MPa^−1^. The other parameters are listed in Table 1.

**Table 1:** The detailed parameters of sand packs in three experiments.

	Grains size (mesh)	Porosity (%)	Permeability (μm^2^)	Connate water saturation (%)
Experiment A	50–75	34.5	11.4	3.4
Experiment B	50–75	29.8	9.8	5.9
Experiment C	50–75	36.4	12.1	3.2

Two heavy oil samples were prepared for the experiments. The first oil sample (No. 1) was crude oil derived from the MPE-3 Block in Venezuela, and the second oil sample (No. 2) was a mass fraction of 90% Venezuelan crude oil mixed with 10% kerosene. Methane was used as the dissolved gas in these experiments. Under the designated conditions of temperature at 50.5 °C and pressure at 12.2 MPa, the specific parameters of the oil samples are shown in Table 2.

**Table 2:** The specific parameters of the oil samples (50.5 °C, 12.2 MPa).

Parameters	No. 1	No. 2
Viscosity (mPa · s)	1518.0	507.1
Density (g/cm^3^)	0.972	0.961
Volumetric factor (cm^3^/cm^3^)	1.0605	1.0559
Dissolved gas-oil ratio (GOR) (cm^3^/cm^3^)	17.50	17.50

According to the formation water properties, the water used in the experiments was a NaHCO_3_ type in which the total salinity was 12,500 ppm, the concentrations of HCO^−^_3_ and Cl^−^ were 2,450 ppm and 10,350 ppm, respectively, and the PH value was 7.35–7.75.

### Experimental procedures

Three depletion experiments, denoted as experiments A, B, and C, were carried out under conditions of a fixed temperature of 50.5 °C and an initial pressure of 12.2 MPa (reservoir temperature and pressure). Oil sample No. 1 was used in both experiments A and B with depletion rates of 0.4 MPa/min and 0.2 MPa/min, respectively, whereas sample No. 2 was used only in experiment C with a depletion rate of 0.2 MPa/min. Bubble-point pressure and pseudo-bubble-point pressure are determined by depletion experiments. The bubble-point pressure of both oil samples is 8.45 MPa; the pseudo-bubble-point pressures in experiment A, B, and C are 4.68 MPa, 4.92 MPa, and 5.01 MPa, respectively.

The experimental procedures were conducted as follows:

1. Before starting the experiments, the sand pack was initialized from vacuum to the designated pressure and then saturated with brine water. The saturated water volume was measured for determining the porosity. The absolute permeability was measured by injecting water at different rates through the sand pack.
2. Live oil (the oil contains dissolved gas) was prepared in the sample container by mixing the methane gas and dead oil to the desired solution gas-oil ratio (GOR) at the designated pressure. Prior to mixing, 5–10 mm diameter glass beads were placed in the sample container to accelerate the dissolution of gas in the oil. The inlet was closed after injection and the mixture was stirred at a fixed rate at constant temperature for about 10 to 30 days to ensure homogeneous dissolution of the gas in the crude oil.
3. The intermediate container was initialized from vacuum to the designated pressure and the saturated oil was subsequently transferred thereto at high pressure. The oil sample was injected from intermediate container into the sand pack from the inlet end at a rate of 1.0 ml/min until the sand-pack pressure reached the desired value. Then, the oil and irreducible water saturation were calculated. The sand pack was allowed to equilibrate for at least 24 hours before the experiment was initiated.
4. During each depletion experiment, the valve at the injection end was kept closed while the pressure at the production end was decreased at a constant rate under the control of the back pressure pump. The cumulative oil and gas productions were measured at regular time intervals. Each experiment was halted when the outlet did not produced oil any more.
5. The oil recovery was the result of the cumulative oil production divided by the total oil within the sand pack, whereas the production GOR was the result of the cumulative gas production divided by the cumulative oil production.

## Mathematical Models

**Figure 2:**
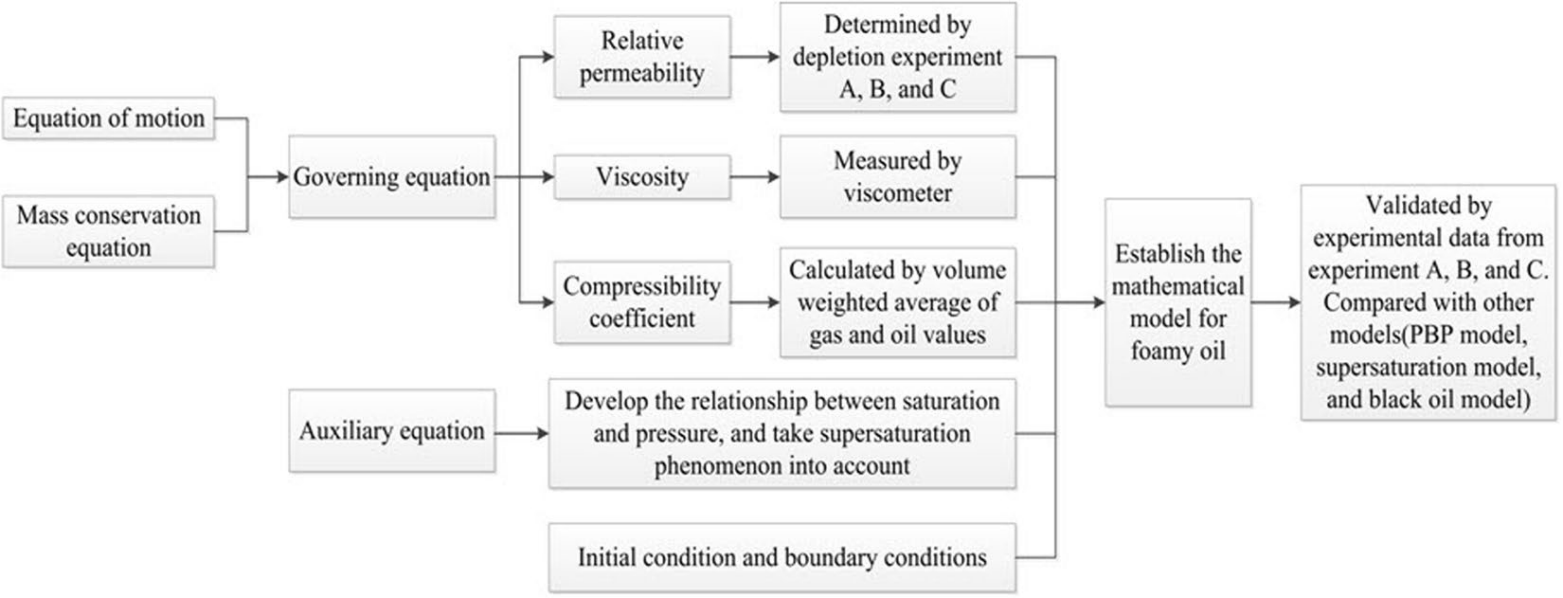
Workflow to illustrate the process of developing the mathematical model for foamy oil.

In this section, a mathematical model will be established to simulate the development of foamy oil reservoirs. To understand to whole process easily, a workflow was made to illustrate how we develop this model in Fig. 2.

### Assumptions

1. Only oil and gas phases are present, and foamy-oil flow follows Darcy’s law under reservoir conditions. Fluid flow is isothermal.
2. All released solution gas remains entrained in the oil phase until the reservoir pressure drops to the pseudo-bubble-point pressure. Below the pseudo-bubble point pressure, free gas begins to form.
3. The effects of the capillary force acting between liquid and gas phases, and gravity are both ignored.
4. The liquid phase is slightly compressible while the gas phase is strongly compressible.

### Determination of the key parameters of the model

#### The effective permeability in porous media

Because foamy oil is regarded as a pseudo-single phase, and the dispersed gas bubbles are viewed as a part of the oil phase. The bubbles are assumed to flow with the oil^23^. Devereux (1974) assumed that the permeability of a porous media decreases as the fraction of droplets in the emulsion increases, and implied that the permeability reduced with decreasing flow rate and with an increasing drop-size to pore-size ratio^37^. Soo and Radke (1986) also proposed a similar theory^38^. These theories are suitable for foamy oil as well, where the high pressure depletion rate causes that the large bubbles in the oil are divided into smaller bubbles; thus, the higher the pressure depletion rate, the less the permeability reduces^39^. A higher crude oil viscosity ensures that the bubble diameter can be stabilized at a smaller scale, corresponding to a less influence on the permeability reduction in porous media^23, 40, 41^.

Based on the above analysis, the volume fraction of the dispersed bubbles (or gas saturation), viscosity of crude oil, and the pressure depletion rate have a significant influence on the permeability in porous media. The relationship between the effective permeability of sand pack and above influence parameters will be built in the subsequent process^42–46^.

During the depletion processes of experiments A, B, and C, the redefined effective permeability of the sand pack was measured at different outlet pressure when the pressure was less than the pseudo-bubble-point pressure. When the outlet pressure equals pseudo-bubble-point pressure, the gas saturation is called critical gas saturation (CGS), which is defined as the minimum gas saturation at which gas-phase flow can occur.

Here, redefined relative permeability *K*_*r*_ is defined as

1 $${K}_{r}=\frac{{K}_{eff}}{K}{\rm{.}}$$

Where, *K*_*r*_ is dimensionless permeability, decimal; *K* is absolute permeability, μm^2^; *K*_*eff*_ is the effective permeability of foamy-oil-flow in the porous media, μm^2^.

**Figure 3:**
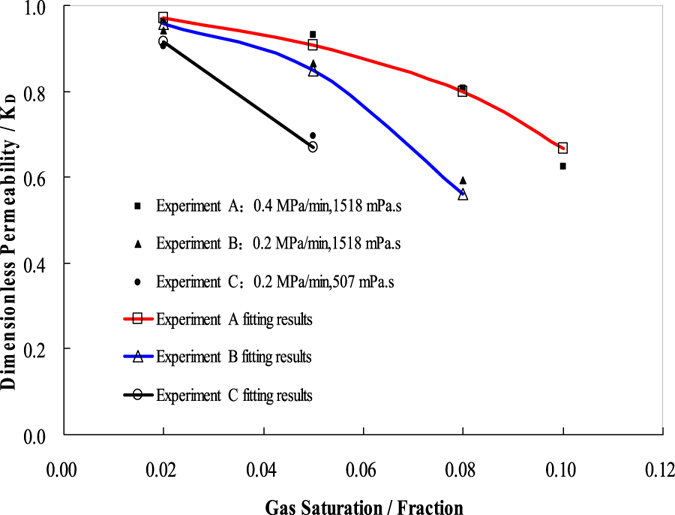
Redefined relative permeability as a function of gas saturation for the three experiments conducted under different conditions.

Figure 3 shows the relationship between redefined relative permeability and gas saturation based upon the results measured of the above three depletion experiments (experiments A, B, and C).

The redefined effective permeability was calculated according to Darcy’s law. The gas saturation was calculated by the Henry’s law and the real gas state equation. Henry’s law means the dissolved gas oil ratio increases linearly with the increase of pressure, especially in heavy oils, this regulation is much more accurate^47–49^. Therefore, the gas saturation near the outlet end was obtained according the outlet pressure, and the redefined effective permeability was computed by the pressure gradient, fluid viscosity, and the flow velocity at the outlet end. Notably, the redefined effective permeability and gas saturation in Fig. 3 represented the corresponding values at outlet ends of sand packs in three experiments. The results of these three experiments also coincide with the analysis above. The corresponding relative permeability increases with the increase of viscosity of oil sample and depletion rate when the gas saturation is smaller than the critical gas saturation (CGS).

From Fig. 3, if *K*_*r*_ is a function of the pressure depletion rate, oil viscosity, and gas saturation when the gas saturation is less than CGS, the oil viscosity and pressure depletion rate are in proportion to *K*_*r*_, and the gas saturation is in inverse proportion to *K*_*r*_. To clarify, we define a dimensionless parameter *β*:

2 $$\beta =\alpha \,\frac{{p}_{d}\cdot {\mu }_{o}}{{S}_{g}}\cdot ({S}_{gc}-{S}_{g}).$$

Where: *β* is a dimensionless parameter, dimensionless; *α* is unit conversion factor, 6 × 10^10^/MPa^2^; *p*_*d*_ is pressure depletion rate, 10^−1^ MPa/min; *μ*_*o*_ is the oil viscosity, mPa · s; *S*_*g*_ is the gas saturation, decimal; *S*_*gc*_ is the gas critical saturation, decimal.

As shown in Fig. 3, *K*_*r*_ increasingly approaches a value of 1 as the value of *β* increases. As such, *K*_*r*_ can be expressed as

3 $${K}_{r}={\rm{In}}(1+\frac{1}{\chi \cdot \beta }{)}^{\chi \cdot \beta }$$

Where, *K*_*r*_ is relative permeability, dimensionless; *χ* is a constant which need experimental fitting, dimensionless.

It can be proven that, when *χ*·*β* tends to infinity, the value of *K*_*r*_ approaches 1, indicating that the effective permeability of foamy-oil flow in the sand pack approaches that of the absolute permeability. The value of *χ* can be obtained from the experimental results through trial and error. Thus, the CGS and *χ* obtained in experiments A, B, and C are shown in Table 3.

**Table 3:** The critical gas saturation (CGS) and *χ* obtained in experiments A, B, and C.

	Experiments A	Experiments B	Experiments C
CGS	0.129	0.094	0.072
*χ*	0.005	0.01	0.02

#### The viscosity of foamy oil

The apparent viscosity of the foamy oil is an important parameter in modeling the behavior of foamy-oil flow. Application of emulsion theories suggests that the foamy dispersion viscosity should be higher than the continuous phase viscosity^38, 49^. However, such theories, when concerned with foamy-oil flow applied directly to porous media, remain questionable^22^. Researchers have evaluated foamy oil viscosity using different methods, where the results are higher or lower than saturated oil viscosity under equivalent conditions^2, 50–55^.

Alshmakhy and Maini used three different viscosity measurement techniques to measure the apparent viscosity of foamy oil, saturated oil, and dead oil^55^. The researchers found the viscosity of foamy oil to be similar to that of saturated oil over a large range of gas saturation. In the present paper, it is assumed that the viscosity of foamy oil is equal to that of saturated oil under equivalent conditions, and this assumption also fitted well with the experimental measure. So the saturated oil viscosity is a function of the pressure *p*, the foamy oil viscosity is therefore obtained by the saturated oil viscosity at different pressures according to the following relationship:

4 $${\mu }_{fo}={\mu }_{o}(p)$$

Where, *μ*_*fo*_ is foamy-oil viscosity, mPa · s.

#### The compressibility coefficient of foamy oil

The compressibility coefficient of foamy oil is composed of two parts–the oil and the gas compressibility coefficients^5^. It complies with the linear volume weighted average compressibility coefficients of oil and gas when the gas concentration is much higher or lower than that of oil^56^. According to the data in above three experiments, the maximum gas saturation was less than 12% when the foamy-oil phenomenon occurred. Therefore, the value of the foamy-oil compressibility coefficient *c*_*fo*_, can be expressed as

5 $${c}_{fo}={S}_{o}{c}_{o}+{S}_{g}{c}_{g}$$

where, *c*_*g*_ at a temperature *T* is given as

6 $${c}_{g}=\frac{1}{p}-\frac{1}{Z}{(\frac{\partial Z}{\partial p})}_{T}$$

The total compressibility coefficient *c*_*t*_ is the sum of the rock compressibility coefficient *c*_*f*_ and *c*_*fo*_:

7 $${c}_{t}={c}_{f}+{c}_{fo}$$

where, *c*_*fo*_ is the compression coefficient of foamy oil, MPa^−1^; *c*_*o*_ is the compression coefficient of oil phase, MPa^−1^; *c*_*g*_ is the compression coefficient of gas phase, MPa^−1^; *c*_*t*_ is the total compression coefficient, MPa^−1^; *c*_*f*_ is the compression coefficient of rock, MPa^−1^; *S*_*o*_ and *S*_*g*_ are the saturation of oil and gas, respectively, decimal; *Z* is the compressibility factor, dimensionless; *T* is temperature, K; *p* is the pressure, 10^−1^ MPa.

### Derivation of the mathematical model

#### Modeling the fluid flow equation

Emulsion has been typically treated as a single phase in previous studies^37, 38^. Similarly, for foamy-oil flow, foamy oil is herein treated as a pseudo-single phase. The mass conservation equation can be expressed as follows:

8 $$\nabla \cdot ({\rho }_{fo}{v}_{fo})=-\frac{\partial (\varphi {\rho }_{fo})}{\partial t}$$

According to the assumptions, foamy-oil flow conforms to Darcy’s law:

9 $${v}_{fo}=-\frac{{K}_{eff}}{{\mu }_{fo}}\cdot \nabla p$$

Combining Eq. (8) and Eq. (9), and employing *c*_*t*_ yields

10 $${\nabla }^{2}p+{c}_{fo}{(\nabla p)}^{2}=\frac{\varphi {\mu }_{fo}{c}_{t}}{{K}_{eff}}\cdot \frac{\partial p}{\partial t}$$

where, *ρ*_*fo*_ is the density of foamy oil, g/cm^3^; *v*_*fo*_ is the velocity of foamy oil, cm/s; *ϕ* is the porosity of porous media, decimal; ∇*p* is the pressure gradient, 10^−1^ MPa/cm; *t* is time, s.

Eq. (10) is the general formula of foam oil flow in heavy oil reservoirs. Actually, the conventional black oil model is its specific expression.

1. When the reservoir pressure is higher than bubble-point pressure, *K*_*eff*_ becomes the absolute permeability *K*, *μ*_*fo*_ becomes *μ*_*o*_, and the oil compressibility coefficient is very small, the second term on the left side of Eq. (10) can be ignored. The model can be simplified as conventional black oil model.
2. When the reservoir pressure is between bubble-point pressure and pseudo-bubble-point pressure, the order of magnitude of the foamy-oil compressibility coefficient is very large and closed to that of gas, and, therefore, the second term on the left side of Eq. (10) cannot be ignored. It represents foamy oil flow in heavy oil reservoirs.
3. When the reservoir pressure is lower than pseudo-bubble-point pressure, it is the oil-gas two-phase flow; the model becomes dissolved gas-drive process in black oil model^57, 58^.

In Eq. (10), *K*_*eff*_ is given by Eq. (1) and Eq. (3), *μ*_*fo*_ is given by Eq. (4), and *c*_*fo*_ and *c*_*t*_ are given by Eq. (5) and Eq. (7), respectively. In Eq. (10), the two variables *p* and *S*_*g*_ (from Eq. (5)) require an auxiliary equation to establish their relationship.

#### Establishment of the auxiliary equation

During the process of foamy-oil flow stage, the production gas-oil ratio (GOR) is approximately equal to the solution GOR. Therefore, the velocities of the dispersed bubbles and the oil are equivalent, and the amount of total gas in the control volume (solution gas plus free gas) is a constant. Based on this fact, an equation of state has been derived^28^. It is supposed that, under a given pressure and temperature, a sealed control volume contains an oil volume *V*_*o*_ and a free gas volume *V*_*g*_, given as

11 $${V}_{g}=\frac{{S}_{g}}{(1-{S}_{g})}\cdot {V}_{o}$$

where, *V*_g_ is free gas volume, cm^3^; *V*_*o*_ is oil volume, cm^3^_._

According to Henry’s Law, the amount of solution gas in the control volume, given as the solution GOR *R*_*si*_, is proportional to the pressure:

12 $${R}_{si}=k(p-{p}_{sc})$$

Eq. (12) can be expressed as another form

13 $${R}_{si}={V}_{gd}^{sc}/{V}_{o}^{sc}$$

where, the subscript ‘*sc*’ refers to the volume at the standard pressure (0.1 MPa) and temperature (20 °C). *V*_*gd*_ is the volume of gas that the system drops from a certain pressure and temperature to the standard state; *V*_*o*_ is the oil phase volume at the standard state, cm^3^.

The real gas law is applicable; the number of moles of the gas can be expressed as

14 $$N=p{V}_{g}/ZRT$$

where, *N* is the number of moles of the gas, mol; *R* is gas constant, J/ (mol · K), given as 8.314.

Under any pressure and temperature conditions, the sum of the number of moles of gas bubbles and solution gas equals the total moles of gas. Applying the standard pressure and temperature, and replacing *V*_*g*_ by *V*_*gd*_, Eq. (14) can be used to calculate the number of solution gas moles at the bubble point pressure *p*_*b*_, and using Eqs. (12) and (13) below the bubble point, yields

15 $$\frac{{p}_{sc}{V}_{o}^{sc}}{R{T}_{sc}}\cdot k({p}_{b}-p)=\frac{p{V}_{g}}{ZRT}$$

where, *p*_d_ is pressure depletion rate, 10^−1^ MPa/min; *p*_b_ is bubble point pressure, 10^−1^ MPa; *T*_sc_ is standard temperature, given as 20 °C.

The definition of volumetric factor *B*_*o*_ is:

16 $${B}_{o}={V}_{o}/{V}_{o}^{sc}$$

where, *B*_o_ is volumetric factor, fraction.

Substituting Eq. (11) into Eq. (15) and using *B*_*o*_, the following equivalent equation of solubility is thereby obtained:

17 $$\frac{{p}_{b}-p}{p}=\gamma \cdot \frac{{S}_{g}}{1-{S}_{g}}$$

where, is a solubility factor, dimensionless; which is given by

18 $$\gamma =\frac{{B}_{o}}{Zk{p}_{sc}}\cdot \frac{{T}_{sc}}{T}$$

Eq. (17) gives the relationship between *S*_*g*_ and *p*, and, when *p* equals the pseudo-bubble point pressure, it can be used to calculate the CGS. In fact, when *p* decreases, the foamy-oil supersaturation phenomenon always occurs, which means the solution gas in the oil phase is more than the value calculated by equilibrium thermodynamics. However, Eq. (17) does not take into account the supersaturation phenomenon^36^. With the generation of bubbles, the system gradually is restored to a balanced state^12^. The dimensionless pressure and dimensionless gas saturation can be defined as follows.

19 $${p}^{\ast }=\frac{{p}_{b}-p}{p},\,{S}_{g}^{\ast }=\gamma \frac{{S}_{g}}{1-{S}_{g}}$$

When the supersaturation phenomenon was taken into account, the two dimensionless parameters were subtracted to define the following supersaturation equation^36^.

20 $$f({p}^{\ast },{S}_{g}^{\ast })={p}^{\ast }-{S}_{g}^{\ast }$$

Where, p* is dimensionless pressure, dimensionless; *S*_g_* is dimensionless gas saturation, dimensionless.

We consider the case where the system pressure is suddenly reduced from *p*_*b*_ to a lower value. Because the formation of bubbles requires a finite length of time, *S*_*g*_ = 0 at the beginning, and, hence, *S*_g_^*^ remains initially zero while p* > 0 of Eq. (20). Therefore, if *f* (p*, *S*_*g*_ *) is positive, the system is supersaturated. The supersaturation pressure decreases as time increases, whereas the rate of decrease is proportional to the supersaturation and inversely proportional to the time; thus, the process can be represented by

21 $$\frac{\tau }{{(1-{S}_{g})}^{2}}\cdot \frac{\partial {S}_{g}}{\partial t}={p}^{\ast }-{S}_{g}^{\ast }$$

where, *τ* is the time required to transform from a non-thermodynamic equilibrium state to a thermodynamic equilibrium state, s. It varies with the properties of the crude oil and the relevant operating conditions, which can be determined by experiments.

When the system is in a supersaturated state, the right side of Eq. (21) is a positive value, where the gas saturation begins to rise, and Eq. (21) describes the variation of the foamy-oil flow process with respect to time. Eq. (21) and Eq. (10) together with the initial and boundary conditions can constitute solvable partial differential equations, which represent the new mathematical model for foamy-oil flow. The corresponding initial conditions are,

22 $$p(x,t=0)={p}_{i}$$

and the boundary condition is

23 $$p(x=L,t)=p(t)$$

where, *L* is location of the sand pack, cm; *p*_i_ is initial pressure in sand pack, 10^−1^ MPa.

### Solving method

If the reservoir pressure is greater than the bubble-point pressure and less than the pseudo-bubble-point pressure, the black oil model is employed for these two cases. When the pressure is higher than the bubble-point pressure, It is very easy to obtain numerical solution by the finite-difference method to solve Eq. (10) (the second term on the left side of Eq. (10) can be ignored). When the pressure is lower than the pseudo-bubble-point pressure, the model becomes dissolved gas-drive process in black oil model. To solve this stage of the model, it is necessary to establish two equations of oil and gas, and the relation between these two equations is that gas saturation plus oil saturation equal unit. For gas equation, Perrine-Martin approximation can be used to address the square term of the pressure derivative. Therefore, the model can be solved by coupling gas and oil equations.

If the reservoir pressure is between the bubble-point pressure and pseudo-bubble-point pressure, the new model can be employed for this case. Eq. (10) can be numerically solved by an expression transformation of ${c}_{fo}p=\,\mathrm{ln}\,p^{\prime}$ just like the method of solving the black oil model. The pressure and gas saturation at each time step can be obtained simultaneously by combining Eq. (10), Eq. (17), and Eq. (21) through using numerical solution method.

## Results and Discussion

### Experimental results

Measurements of the cumulative oil and gas production were employed to calculate the oil recovery and production GOR, and these two production indicators were used to evaluate the extent of agreement between the models and experiments. The results in experiments A, B, and C are shown in Table 4.

**Table 4:** Depletion experimental results for experiments A, B, and C.

Outlet pressure (MPa)	Experiment A		Experiment B		Experiment C	
	Oil recovery (%)	Production GOR (cm^3^/cm^3^)	Oil recovery (%)	Production GOR (cm^3^/cm^3^)	Oil recovery (%)	Production GOR (cm^3^/cm^3^)
11.6	0.18	17.5	0.21	17.5	0.24	17.5
10.8	0.26	17.5	0.29	17.5	0.32	17.5
10.0	0.38	17.5	0.37	17.5	0.47	17.5
9.2	0.49	17.5	0.56	17.5	0.68	17.5
8.4	0.83	28.9	1.17	17.5	1.29	19.7
8.0	1.35	25.5	2.07	23.4	2.07	23.6
7.6	3.02	31.9	3.16	37.7	2.98	39.4
7.2	4.78	32.6	4.45	31.5	4.54	35.3
6.8	6.97	40.5	6.12	38.9	6.19	38.4
6.4	9.32	37.8	8.16	35.7	7.34	40.1
6.0	11.06	35.9	9.88	41.6	8.53	37.2
5.6	12.14	37.6	11.08	50.5	9.68	45.1
5.2	14.12	41.5	11.77	39.8	10.46	42.9
4.8	15.54	54.7	12.62	79.5	10.87	60.2
4.0	16.30	89.6	13.14	201.6	11.21	109.5
3.2	16.70	262.5	13.41	269.7	11.53	279.3
2.4	16.88	443.8	13.68	428.6	11.79	417.7

Note: bubble-point pressure for both oil samples is 8.45 MPa, and pseudo-bubble-point pressures for experiment A, B, and C are 4.68 MPa, 4.92 MPa, and 5.01 MPa.

### Model validation

The depletion experiment of the sand pack can be divided into three stages.

#### The first stage represents single-phase oil flow

In this stage, both the oil recovery and the production GOR are very low. The sand-pack pressure is greater than the bubble point pressure estimated by the black oil model (the model we used is a commercial numerical simulator named as Computer Modelling Group (CMG), which was developed by a Canadian company) in this stage.

#### The second stage is foamy-oil flow

This stage represents the major oil-production stage of the depletion experiment. The sand-pack pressure lies between the bubble point pressure and the pseudo-bubble point pressure. The production GOR was slightly higher than the solution GOR. The foamy-oil recovery was found to increase with the increase of pressure depletion rate and oil viscosity. To better verify the simulation accuracy, the new model, the PBP model^26^, the supersaturation model^36^, and the conventional black oil model (CMG) were used for comparison with the data of experiments A, B and C.

Based on experimental results, the relaxation time *τ* = 6 × 10^5^ s, 4.7 × 10^5^ s, and 4.2 × 10^5^ s in the new model and the supersaturation model for the conditions in experiments A, B, and C, respectively. In the PBP model, the phenomenon of supersaturation was not considered, that is to say, Eq (17), not Eq (21), was used to compute in the model. In order to compare the results more obviously, the black oil model was also employed in this stage.

#### The third stage is the oil-gas two-phase flow

Upon completion of the foamy-oil flow stage, the sand-pack pressure is lower than the pseudo-bubble point pressure, and the gas phase becomes a continuous phase and begins to flow. The oil recovery was low and the production GOR increased sharply, the black oil model (CMG) was used to simulate the production performance in this stage.

### Analysis and discussion

**Figure 4:**
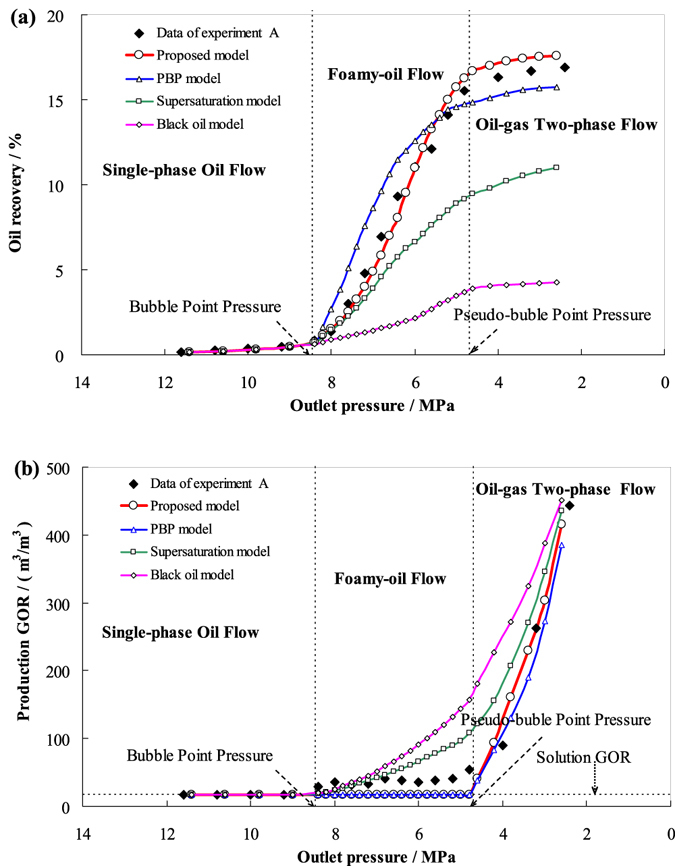
The simulated results by the four models in comparison with the experimental data for experiment A. (**a**) oil recovery with respect to outlet pressure. (**b**) Production GOR with respect to outlet pressure.

**Figure 5:**
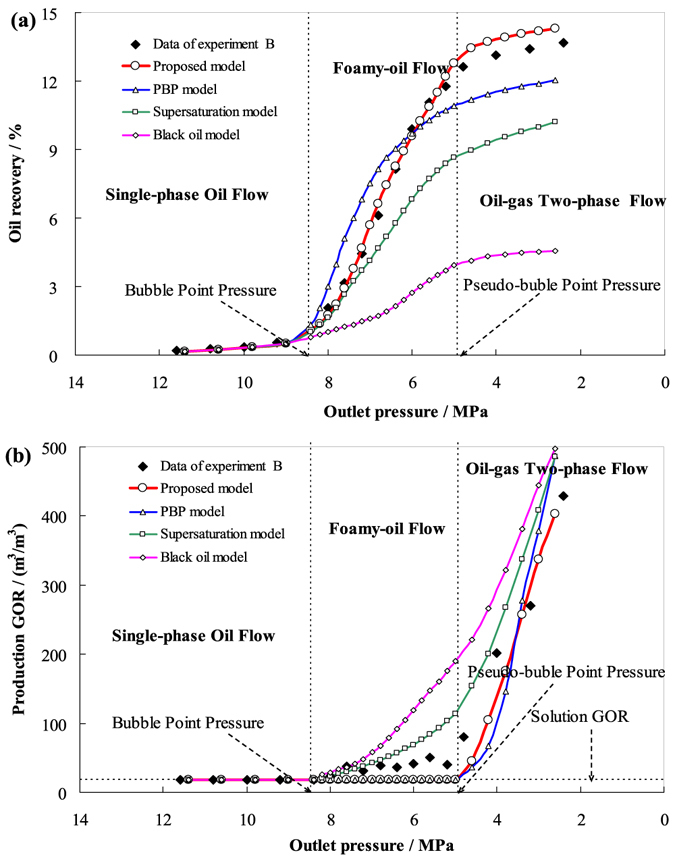
The simulated results by the four models in comparison with the experimental data for experiment B. (**a**) Oil recovery with respect to outlet pressure. (**b**) Production GOR with respect to outlet pressure.

**Figure 6:**
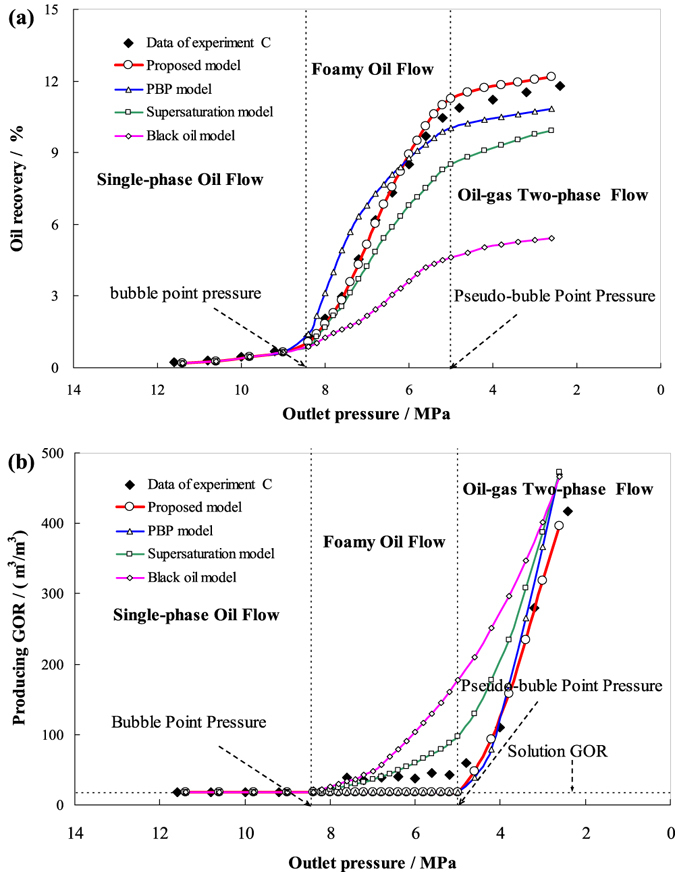
The simulated results by the four models in comparison with the experimental data for experiment C. (**a**) Oil recovery with respect to outlet pressure. (**b**) Production GOR with respect to outlet pressure.

The parameters used to solve the models were obtained from the depletion experimental data. Figures 4, 5, and 6 respectively show the comparison results of the oil recovery and the production GORs simulated by the new model, the pseudo-bubble-point (PBP) model, the supersaturation model, and the black oil model (CMG) with those of the experiments A, B, and C.

From Figs 4(a), 5(a), and 6(a), the oil recoveries simulated by the new model agree well with the experimental data, except in the late stages. This is because in the actual flow process, the possibility of bubble coalescence and the formation of narrowing flow path channels increases with the increase of gas saturation and results in reduced oil mobility, the simulated recoveries are slightly higher than that indicated by the experimental data. However, the PBP model does not take the supersaturation phenomenon into account, so the gas saturation in foamy oil is higher than the real value. It has two opposite effects, on the one hand, high gas saturation increases the system’s compression coefficient; on the other hand, it reduces the effective permeability of the core pack. The result of the comprehensive effect is that the oil recovery is higher than experimental data in the early stage of the foam oil flow and lower than the experimental data in the late stage. Though the supersaturation model takes the supersaturation phenomenon into consideration, it addresses the foamy-oil flow as oil-gas two-phase flow, which makes the mobility of gas higher than real situation, thereby producing lower oil recoveries and higher production GOR than the experimental data. The black oil model does not take the foamy-oil properties into account, and addresses the foamy-oil flow process as conventional oil-gas two-phase flow, which makes simulation results quite different from the experimental data.

From Figs 4(b), 5(b), and 6(b), the simulated production GOR by the new model and PBP model agree well with the experimental data. The reason why production GOR remain low levels when the pressure higher than pseudo-bubble-point pressure is that the interfacial tension between heavy oil and gas are too strong, which makes the heavy oils capture and restrain the gas as separate gas bubbles and cannot form continuous free gas channel immediately^59^. Finally, free gas will form when the pressure drops to pseudo-bubble point and production GOR will soar to a big value. However, the results obtained from the supersaturation model and the black oil model indicated that the production GOR increased significantly when the pressure was below the bubble point pressure, especially the black oil model (CMG), which is inconsistent with the experimental results.

Taken together, it is readily apparent that the experimental data is in better agreement with the results simulated by the new model than the other three models, which represents we obtain progress in simulating and evaluating foamy oil’s performances and characteristics on foamy-oil flow in porous media for heavy oil reservoirs.

## Conclusions

Based on the mathematical model proposed and experimental validation in this paper for foamy-oil flow in porous media for heavy oil reservoirs, the following conclusions can be drawn, and the underlying drawbacks of this model are also pointed.

1. According to the depletion experimental results, the redefined effective permeability of the sand pack varying with the pressure depletion rate, oil viscosity, and gas saturation, and an appropriate mathematical expression for the effective permeability on foamy-oil flow in porous media was developed.
2. It was assumed that the foamy-oil viscosity is equal to the saturated-oil viscosity under equivalent conditions, which was justified by the results of the model.
3. The compressibility coefficient of foamy oil is a volume-weighted of the oil and gas phase compressibility coefficients. The foamy-oil volume dramatically expands between the bubble point pressure and the pseudo-bubble point pressure, which allows significant displacement of the oil.
4. The particular fluid properties of foamy oil are considered the main cause of high oil recovery during the depletion process in heavy oil. The second stage of foamy-oil flow is the main oil-production stage, where high oil recovery is obtained and production GOR is slightly higher than solution GOR.
5. By taking into account foamy-oil supersaturation, a mathematical model that can reflect foamy-oil characteristics was modeled. As verified by three depletion experiments, the simulation results of the new model agree well with the experimental data, while the results obtained from the pseudo-bubble-point (PBP) model, the supersaturation model, and the black oil model (CMG) were not satisfactory. These results suggest that the new model is suitable for application for foamy-oil flow in porous media of heavy oil reservoirs.
6. The determination of some parameters in the model needs experimental assistance, like pseudo bubble point pressure and relaxation time, which requires relatively high accuracy of experiments. High experimental errors may cause wrong results. In addition, the model was only verified by sand pack experiments, therefore, whether it is suitable for reservoir development or not still needs further study.
